# Circulating microbiome in patients with portal hypertension

**DOI:** 10.1080/19490976.2022.2029674

**Published:** 2022-02-07

**Authors:** Rolandas Gedgaudas, Jasmohan S Bajaj, Jurgita Skieceviciene, Greta Varkalaite, Gabija Jurkeviciute, Sigita Gelman, Irena Valantiene, Romanas Zykus, Andrius Pranculis, Corinna Bang, Andre Franke, Christoph Schramm, Juozas Kupcinskas

**Affiliations:** a Department of Gastroenterology, Lithuanian University of Health Sciences, Kaunas, Lithuania; b Institute for Digestive Research, Lithuanian University of Health Sciences, Kaunas, Lithuania; c Department of Internal Medicine, Division of Gastroenterology, Hepatology, and Nutrition, Virginia Commonwealth University and Central Virginia Veterans Healthcare System, Richmond, Virginia, USA; d Department of Radiology, Lithuanian University of Health Sciences, Kaunas, Lithuania; e Institute of Clinical Molecular Biology, Christian-Albrechts-University of Kiel, Kiel, Germany; f Ist Department of Medicine, University Medical Center Hamburg-Eppendorf, Hamburg, Germany; g Martin Zeitz Center for Rare Diseases, University Medical Center Hamburg-Eppendorf, Hamburg, Germany

**Keywords:** Blood microbiome, gut-liver axis, permeability, inflammation, hepatic venous pressure gradient

## Abstract

Portal hypertension (PH) in liver cirrhosis leads to increased gut permeability and the translocation of bacteria across the gut–liver axis. Microbial DNA has recently been detected in different blood compartments; however, this phenomenon has not been thoroughly analyzed in PH. This study aimed to explore circulating bacterial DNA signatures, inflammatory cytokines, and gut permeability markers in different blood compartments (peripheral and hepatic veins) of patients with cirrhosis and PH. The 16S rRNA blood microbiome profiles were determined in 58 patients with liver cirrhosis and 46 control patients. Taxonomic differences were analyzed in relation to PH, liver function, inflammatory cytokines, and gut permeability markers. Circulating plasma microbiome profiles in patients with cirrhosis were distinct from those of the controls and were characterized by enrichment of *Comamonas, Cnuella, Dialister, Escherichia/Shigella*, and *Prevotella* and the depletion of *Bradyrhizobium, Curvibacter, Diaphorobacter, Pseudarcicella*, and *Pseudomonas*. Comparison of peripheral and hepatic vein blood compartments of patients with cirrhosis did not reveal differentially abundant taxa. Enrichment of the genera *Bacteroides, Escherichia/Shigella*, and *Prevotella* was associated with severe PH (SPH) in both blood compartments; however, circulating microbiome profiles could not predict PH severity. *Escherichia/Shigella* and *Prevotella* abundance was correlated with IL-8 levels in the hepatic vein. In conclusion, we demonstrated a distinct circulating blood microbiome profile in patients with cirrhosis, showing that specific bacterial genera in blood are marginally associated with SPH, Model for End-Stage Liver Disease score, and inflammation biomarkers; however, circulating microbial composition failed to predict PH severity.

## Introduction

Portal hypertension (PH) is a common hemodynamic abnormality in cirrhosis that is associated with the development of severe complications, including variceal bleeding, ascites, and hepatic encephalopathy.^1,2^ PH leads to increased gastrointestinal permeability, translocation of bacteria, and endotoxin levels and is associated with infection risk, which remains the major cause of mortality for patients with cirrhosis.^3–5^ Diminished clearance by cirrhotic liver and portosystemic shunting can further activate inflammatory cascades, both in the liver and systemically.^6,7^

Overwhelming evidence indicates the gut microbiome plays a significant role in liver diseases.^8–10^ Interestingly, studies have shown that microbial DNA can be detected in human blood with circulating cell-free DNA analysis identifying numerous highly divergent microbes.^11,12^ Over the last years, several attempts have been made to define changes in the circulating microbiome of patients with liver diseases.^13–15^ For instance, a Japanese study demonstrated that circulating *Enterobacteriaceae* levels are significantly higher in patients with cirrhosis.^15^ In addition, alcohol consumption has been shown to be the primary driver of changes in the circulating microbiome of patients with alcoholic hepatitis.^14^ The level of circulating bacterial DNA also significantly increases in hepatitis B-related acute-on-chronic liver failure.^13^ More recently, circulating microbiome profiles have been explored in several other conditions, including chronic kidney disease,^16^ rheumatoid arthritis, and cancer.^16–18^

Invasive hepatic venous pressure gradient (HVPG) measurements currently remain the gold standard method for assessing portal pressure in patients with liver cirrhosis.^1^ However, the potential value of the circulating microbiome for prediction of PH has not been previously reported.

Due to the presence of shunts that develop with liver cirrhosis, blood from mesenteric veins directly enters the systemic circulation, avoiding the liver barrier.^1^ Furthermore, endotoxins in healthy individuals are cleared from portal blood by Kupffer cells; however, liver injury that occurs in cirrhosis results in leakage and higher endotoxin levels in the hepatic and peripheral circulations.^7^ Endotoxemia, with or without viable bacterial translocation, is a common event in cirrhosis.^19^ Several studies have reported altered endotoxin levels in the portal circulation versus those in the peripheral circulation, indicating a potential intestinal origin for these bacterial products.^7^ Different levels of inflammatory cytokines have also been shown in systemic, portal, and hepatic circulation.^7,20^ A small recent study by Schierwagen et al.^21^ reported distinct microbiome compositions in different blood compartments of patients with cirrhosis; however, further data regarding this are needed. Analysis of the circulating microbiome in different blood compartments in patients with liver cirrhosis is important for several reasons. First, it is important to determine whether the severity of disease is linked to the reduced ability of the liver to clear microbes in hepatic blood outflow. Second, it is important to understand if the degree of PH is associated with circulating microbiome alterations as higher levels of PH may lead to increased microbial burden in the peripheral circulation due to shunting and increased gut permeability.^21^

Our aim in the current study was to use 16S rRNA sequencing of plasma specimens to determine the circulating microbiome signatures in different blood compartments of patients with cirrhosis and PH. We also wanted to determine whether circulating bacterial DNA levels and specific bacterial taxa were correlated with the degree of PH, liver function tests, inflammatory cytokines, and gut permeability markers. In addition, we aimed to assess whether the circulating microbiome profile in peripheral blood could be used as a noninvasive biomarker for the prediction of PH severity.

## Results

### Circulating blood microbiome composition in healthy control individuals and patients with cirrhosis

**Figure 1.**
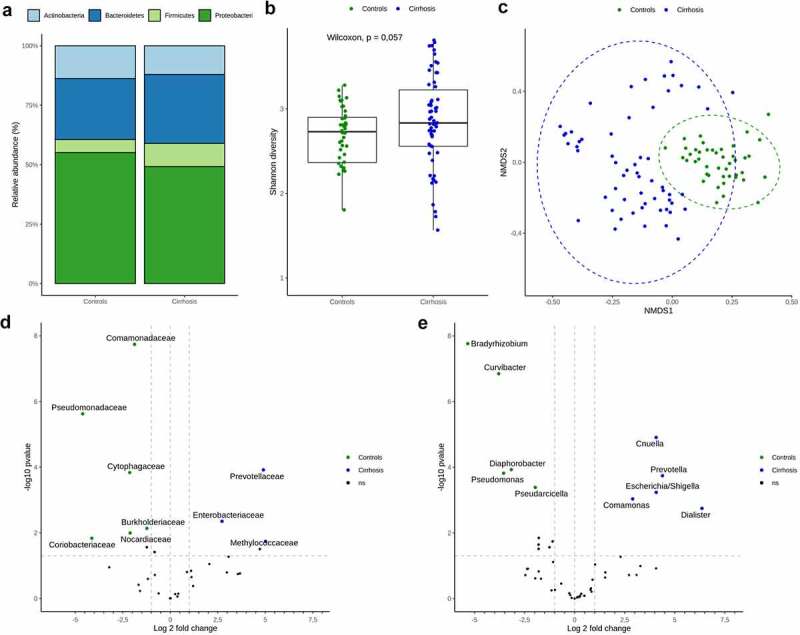
Circulating microbiome comparison between controls and liver cirrhosis patients.

Compositional analysis revealed that the circulating microbiome in the peripheral circulation comprised four phyla in healthy controls and patients with cirrhosis, with *Proteobacteria* being the most dominant, followed by *Bacteroidetes, Actinobacteria*, and *Firmicutes* (Figure 1(a)). The relative abundance of *Firmicutes* was increased in patients with cirrhosis compared to that in healthy individuals (9.7% vs. 5.8%, p = .034), while the relative abundance of *Proteobacteria* (49.2% vs. 55.9%), *Bacteroidetes* (29.1% vs. 25%), and *Actinobacteria* (12% vs. 13.3%) showed no significant differences between the groups. Compositional variation at the phylum level of the circulating microbiome in individual patients with cirrhosis and controls is presented in Supplementary Figures S1 and S2. Bacterial diversity (α-diversity), as assessed by the Shannon diversity index, did not reveal significant differences between patients with cirrhosis and healthy controls (Figure 1(b)). However, significant blood microbial community clusters (β-diversity), as assessed by the Bray-Curtis dissimilarity index, were identified (p < .001; Figure 1(c)).

a) Bar plot representing different phylum relative abundances in peripheral blood of liver cirrhosis patients and controls. b) Shannon diversity index (α-diversity) stratified by diagnosis. c) Blood microbiome community composition (β-diversity) assessed by non-multidimensional scaling on Bray-Curtis dissimilarity, with significant clustering according to diagnosis. d and e) Differential relative abundance testing between liver cirrhosis patients and controls: Taxa with significantly different relative distribution on Family (D) and Genus (E) levels are displayed by volcano plots. The x‐axis shows the log2 fold change, y‐axis displays the – log 10 transformed P‐value. Taxa were considered differentially abundant when reaching a Padj <0.05 and absolute log 2-fold change > 1

Bacterial community clustering between patients with cirrhosis and healthy controls could be explained by the significant differences in the circulating microbiome composition. Compared to the controls, patients with cirrhosis showed an increase in the relative abundance of *Enterobacteriaceae, Methylococcaceae*, and *Prevotellaceae* and a decline in abundance of members of the families *Burkholderiaceae, Cytophagaceae, Comamonadaceae, Coriobacteriaceae, Nocardiaceae*, and *Pseudomonadaceae*. At the genus level, patients with cirrhosis had higher relative levels of *Cnuella, Comamonas, Dialister, Escherichia/Shigella*, and *Prevotella* and lower levels of *Bradyrhizobium, Curvibacter, Diaphorobacter, Pseudarcicella*, and *Pseudomonas* (Figures 1(d-e)). These results indicate a distinct circulating microbiome profile for patients with cirrhosis. Abundance levels of the differentially abundant genera in the peripheral veins of patients with cirrhosis and the controls are shown in Supplementary Figure S3.

### Circulating microbiome in different blood compartments of patients with cirrhosis

**Figure 2.**
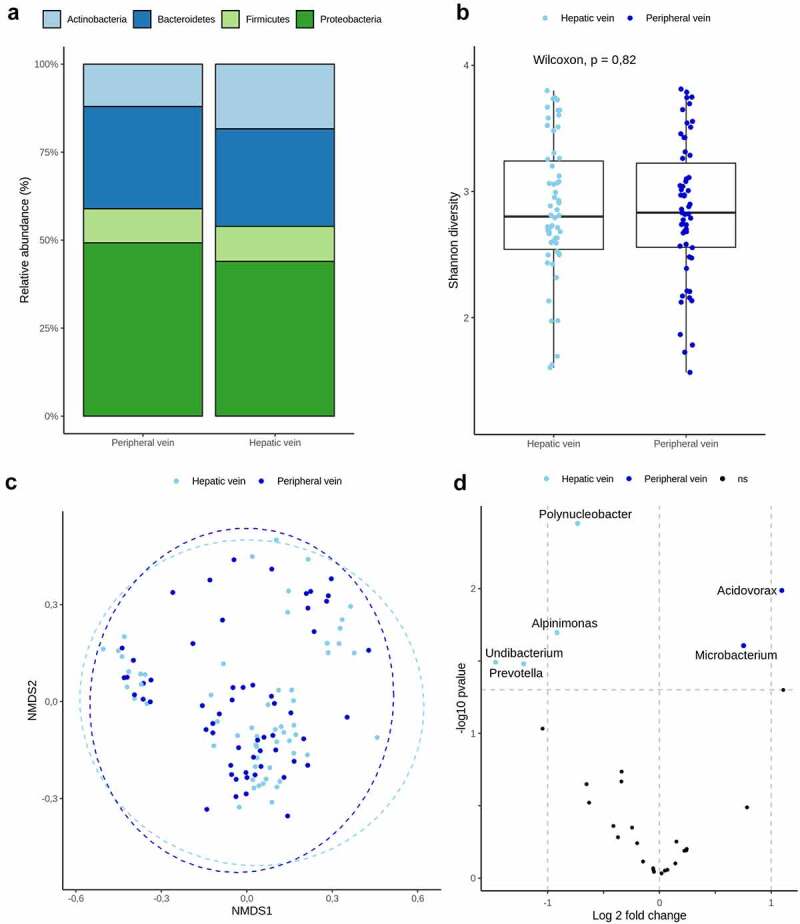
Circulating microbiome in different compartments of liver cirrhosis.

We analyzed the circulating microbiome in the hepatic veins of patients with cirrhosis. The same four phyla of the peripheral circulation microbiome comprised the hepatic vein microbiome, with *Proteobacteria* at 44%, *Bacteroidetes* at 27.7%, *Actinobacteria* at 18.4%, and *Firmicutes* at 9.9% (Figure 2(a)). There were no significant differences in the within-sample diversity (α-diversity) or community structure (β-diversity) between the hepatic vein blood and peripheral vein blood compartments (Figures 2(b-c)). Pairwise differential abundances between the different compartments of the study patients showed a tendency or the genera *Acidovorax* and *Microbacterium* to be enriched in the peripheral veins and *Alpinimonas, Polynucleobacter, Prevotella*, and *Undibacterium* to be enriched in the hepatic veins of patients with cirrhosis; however, these findings did not withstand multiple testing corrections (Figure 2(d)).

a) Bar plot representing different phylum relative abundances in different compartments of liver cirrhosis patients’ blood. b) Shannon diversity index (α-diversity) stratified by different compartments. c) Blood microbiome community composition (β-diversity) between different compartments in cirrhosis assessed by non-multidimensional scaling on Bray-Curtis dissimilarity. d) Differential relative abundance testing between different compartments of liver cirrhosis: Genera with significantly different relative distribution are showed by volcano plots. The x‐axis shows the log2 fold change, y‐axis displays the – log 10 transformed P‐value. Colored are genera with *P < .05*, which, however, did not withstand multiple comparison testing.

### Circulating microbiome and gut permeability

**Figure 3.**
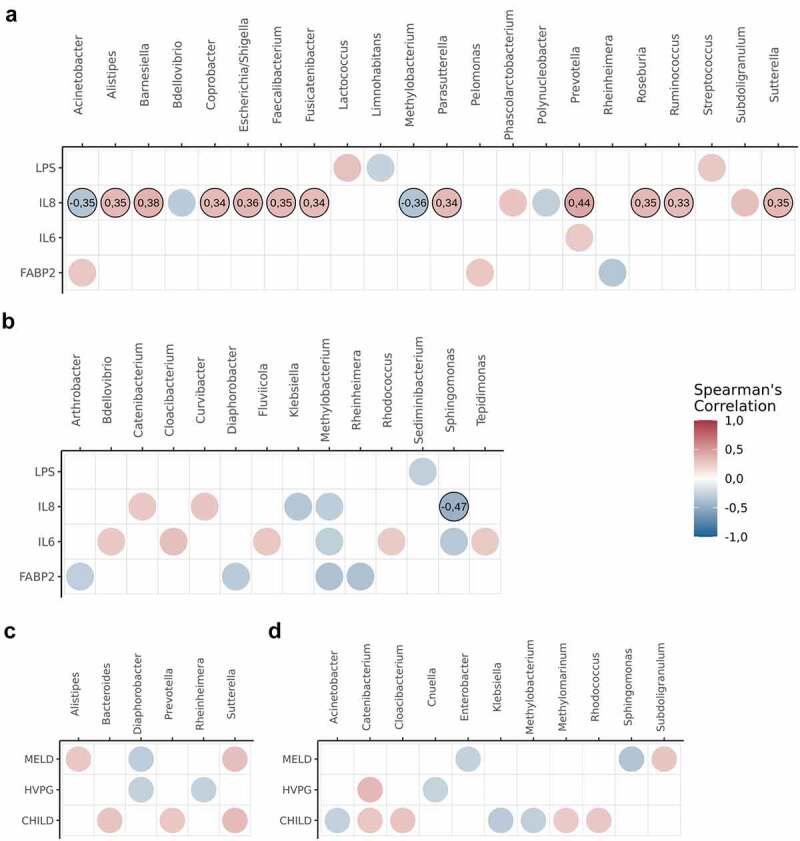
Correlation analysis between circulating microbiome, gut-permeability markers, inflammatory cytokines and clinical parameters of cirrhosis severity.

Expression levels of the gut permeability marker fatty acid-binding protein 2 (FABP2) were higher in the peripheral veins of patients with cirrhosis than in those in the healthy controls (Supplementary Figure S4). Moreover, peripheral levels of FABP2 were correlated with HVPG values (Supplementary Table S1). To assess whether intestinal permeability affected the circulating microbiome composition, we evaluated correlations between FABP2 and the circulating microbiome members; however, none of the genera demonstrated significant associations FABP2 expression (Figures 3(a-b)). Moreover, we did not identify any significant correlations between the circulating taxa and HVPG values in any compartment that withstood multiple comparison testing (Figures 3(c-d)).

a) Spearman’s rank correlation coefficients between abundances of different genera, inflammatory cytokines (LPS, IL-6 and IL-8) and gut permeability markers (FABP2) in hepatic vein blood b) – in peripheral vein blood of patients with cirrhosis. c) Spearman’s rank correlation coefficients between abundances of different genera and cirrhosis parameters in hepatic vein blood; d) – in peripheral vein blood of patients with cirrhosis. Genera are presented on the x-axis, whereas inflammatory cytokines, gut permeability markers and clinical parameters of cirrhosis are presented on the y-axis. Colors represent the Spearman rank correlation coefficients at P value < .05, while numbers – at Benjamini–Hochberg corrected P value < .05. Circles without correlation coefficients did not withstand multiple corrections. Abbreviations: HVPG – hepatic venous pressure gradient; CHILD – Child-Turcotte-Pugh; MELD – Model for End-Stage Liver Disease

### Circulating microbiome and PH severity

**Figure 4.**
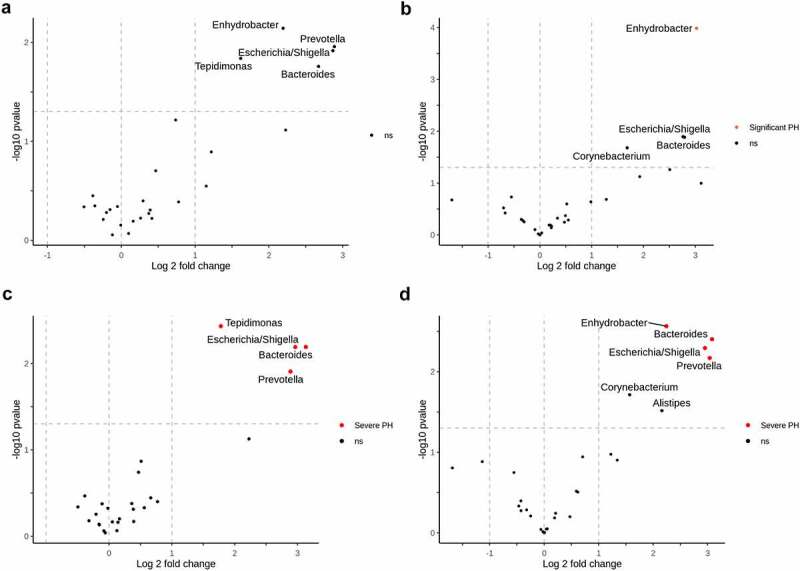
Circulating microbiome and portal hypertension.

**Figure 5.**
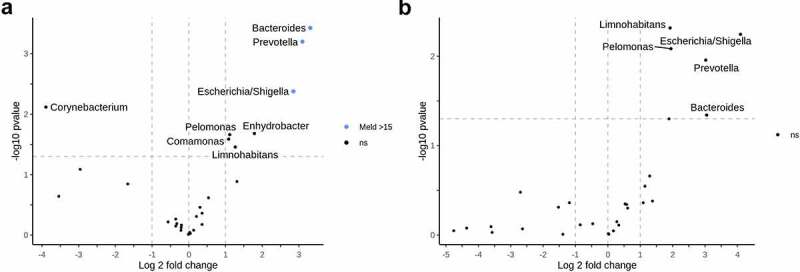
Circulating microbiome and MELD score.

While we did not identify any correlations between HVPG values and the circulating microbiome taxa, we proceeded with differential abundance testing between groups of patients with different PH severity. Most differentially abundant genera failed to withstand multiple comparison testing. However, in the comparison of patients with clinically significant portal hypertension (CSPH) and non-significant PH, *Bacteroides, Escherichia/Shigella, Prevotella*, and *Tepidimonas* in hepatic veins and *Bacteroides, Enhydrobacter, Escherichia/Shigella*, and *Prevotella* in peripheral veins all withstood multiple comparison testing with the genera being more abundant in the patients with severe portal hypertension (SPH) (Figure 4). Moreover, *Bacteroides Escherichia/Shigella*, and *Prevotella* were enriched in both compartments of patients with a Model For End-Stage Liver Disease (MELD) score >15. However, after multiple comparison corrections, the results remained significant only in the hepatic vein blood (Figure 5). Abundances of *Bacteroides, Escherichia/Shigella*, and *Prevotella* in subgroups of patients with different PH severity and MELD scores in the different compartments are shown in Supplementary Figure S5.

Differential relative abundance testing according to the degree of portal hypertension. A volcano plot a) represents genera with significantly different relative distribution between patients with significant (HVPG > 10) and non-significant (HVPG < 10) portal hypertension in hepatic vein blood; b) – in peripheral blood. Plot c) – represents differentially abundant genera between patients with severe (HVPG > 12) and non-severe portal hypertension in hepatic vein blood; d) – in peripheral blood. The x‐axis shows the log2 fold change, y‐axis displays the – log 10 transformed P‐value. Taxa were considered differentially abundant when reaching a *Padj* <0.05 and absolute log 2-fold change >1. Positive log 2-fold changes indicate genera with higher abundance in clinically significant or severe portal hypertension groups. Genera which did not withstand multiple comparison testing are above the horizontal line and marked ns. Abbreviations: HVPG – hepatic venous pressure gradient; PH – portal hypertension, ns – not significant.

Differential relative abundance testing according to the severity of cirrhosis. A volcano plot a) represents genera with significantly different relative distribution between patients with MELD score >15 and <15 in hepatic vein blood; b) – in peripheral blood. The x‐axis shows the log2 fold change, y‐axis displays the – log 10 transformed P‐value. Taxa were considered differentially abundant when reaching a *Padj* <0.05 and absolute log 2-fold change >1. Positive log 2-fold changes indicate genera with higher abundance in patients with MELD score >15. Genera which did not withstand multiple comparison testing are above the horizontal line, non-colored and marked as ns. Abbreviations: MELD – Model for End-Stage Liver Disease, ns – not significant

**Figure 6.**
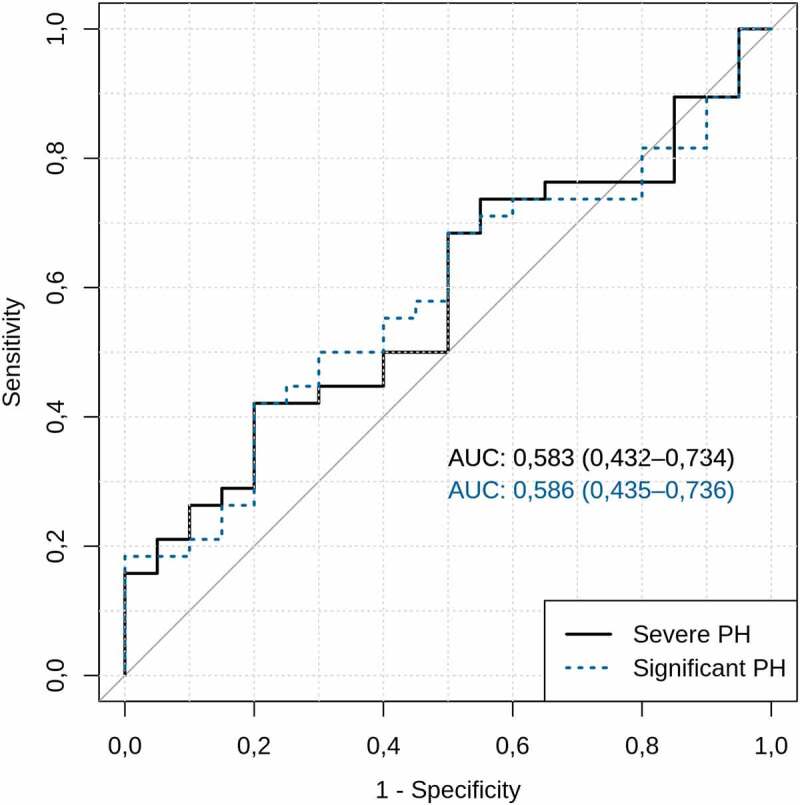
Circulating microbiome and prediction of portal hypertension.

To assess the ability of the circulating microbiome to discriminate patients with CSPH or SPH, we performed receiver operating characteristic (ROC) curve analysis based on the relative abundances of the differentially abundant genera. This sub-analysis revealed no statistically significant differences, giving an area under curve (AUC) of 0.586 (95% CI: 0.435–0.736, p = .44) for CSPH and an AUC of 0.583 (95% CI: 0.432–0.734, p = .17) for SPH, indicating a limited potential for using the circulating microbiome to predict PH in patients with cirrhosis (Figure 6).

Circulating microbiome ability to discriminate patients with clinically significant and severe portal hypertension. Genera used in prediction model were selected based on DESeq2 results: Relative abundances of *Bacteroides+Enhydrobacter+Escherichia/Shigella+Corynebacterium* were used to discriminate patients with clinically significant portal hypertension, while *Prevotella+Bacteroides+Enhydrobacter+Escherichia/Shigella+Corynebacterium+Alistipes* – patients with severe portal hypertension. The values in brackets indicate the 95% confidence interval. AUC – area under the receiver operating characteristic curve; PH – portal hypertension.

### Circulating microbiome and systemic inflammation

To assess the relationship between members of the circulating microbiome and systemic inflammation, we measured the levels of lipopolysaccharide (LPS), interleukin (IL)-6, and IL-8 in both compartments of patients with cirrhosis and in the peripheral veins of the healthy controls. Levels of LPS, IL-6, and IL-8 were higher in patients with cirrhosis than in the healthy controls. Furthermore, IL-6 and IL-8 levels in the peripheral blood were correlated with MELD and Child-Pugh-Turcotte (CTP) scores (Table 1 and Table 2, Supplementary Figure S4). We identified no significant correlations between circulating microbiome genera and cytokines in the peripheral veins; however, the relative abundance of *Alistipes, Barnesiella, Coprobacter, Escherichia/Shigella, Faecalibacterium, Fusicatenibacter, Parasutterella, Prevotella, Roseburia, Ruminococcus*, and *Satturella* was correlated with IL-8 levels in the hepatic vein (Figures 3(a-b)).

**Table 1.** Demographic and clinical characteristics of subject groups

Variable	Controls (n = 46)	Patients (n = 58)	P value
Age, mean ± SD	40.5 ± 14.1	51.6 ± 8	< 0.001
Gender (Female)	36 (75%)	23 (39.7%)	< 0.001
Etiology (Alcohol/HCV)		28/30 (48.3%/51.7%)	
On lactulose		44 (75.9%)	
On PPI		23 (39.7%)	
Parameters of cirrhosis			
CTP class, mean ± SD Class A/B/C		6.7 ± 2.1 62.1%/24.1%/13.8%	
MELD, mean ± SD MELD Score > 15		11.2 ± 4.8 12 (20.7%)	
HVPG, mean ± SD NCSPH/CSPH/SPH		13.1 ± 6.1 20/38/33 (34.5%/65.5%/56.9%)	
Presence of ascites Degree of ascites (Grade 1/Grade 2/Grade 3)		28 (48.3%) 20/8/0 (71.4%/28.6%/0%)	
Presence of esophageal varices (F1/F2/F3)		29 (50%) 15/10/4 (51.7%/34.5%/13.8%)	
Previous history of cirrhosis related events			
History of SBP		3 (5.2%)	
History of variceal bleed		6 (10.3%)	
History of hepatorenal syndrome		2 (3.4%)	
Biochemical tests			
Total/Direct bilirubin, mean ± SD (µmol/l)		33.9 ± 33/12.8 ± 17.5	
Albumin, mean ± SD (g/l)		36 ± 5.9	
INR, mean ± SD		1.3 ± 0.4	
Creatinine, mean ± SD (µmol/l)		74.3 ± 15.7	
Ammonia, mean ± SD (µmol/l)		41.5 ± 17.8	
Cytokine levels			
FABP2 peripheral (pg/ml)	334.5 ± 107	422.9 ± 192.3	0.0382
LPS peripheral (pg/ml)	61.3 ± 46.5	172.3 ± 89.3	< 0.001
IL-6 peripheral (pg/ml)	0.4 ± 0.5	9.1 ± 13.6	< 0.001
IL-8 peripheral (pg/ml)	17.1 ± 3.6	31.4 ± 19.4	< 0.001
FABP2 hepatic vein (pg/ml)		565.8 ± 428.6	
LPS hepatic vein (pg/ml)		99 ± 63	
IL-6 hepatic vein (pg/ml)		9.9 ± 18.9	
IL-8 hepatic vein (pg/ml)		13 ± 19.5	

SD – Standard deviation; HCV – Hepatitis C virus; MELD – Model of End-Stage Liver Disease; HVPG – Hepatic venous pressure gradient; NCSPH – Not clinically significant portal hypertension; CSPH – Clinically significant portal hypertension; SPH – Severe portal hypertension; PPI – Proton pump inhibitors; FABP2 – Fatty acid-binding protein 2; LPS – Lipopolysaccharides; IL- Interleukin.

All of the included patients were on a regular diet. None of the patients with alcohol-induced cirrhosis were active drinkers for at least one month prior to the inclusion in the study. Previous history of cirrhosis-related events represents events in the last six months; none of the included patients had any cirrhosis-related events for at least a month prior to inclusion in the study.

Wilcoxon ranked-sum and Chi square tests were used to compare groups.

**Table 2.** Subgroup characteristics of patients with liver cirrhosis

Variable	HVPG			HVPG			MELD		
	≥12 (n = 33)	<12 (n = 25)	*P v*alue	≥10 (n = 38)	<10 (n = 20)	*P* value	≥15 (n = 12)	<15 (n = 46)	*P* value
Age, mean ± SD	53 ± 7	49.4 ± 8.7	0.125	52.2 ± 7.4	50.3 ± 8.9	0.583	51.5 ± 7.9	51.6 ± 8	0.992
Gender (Female)	17 (51.5%)	6 (24%)	0.034	18 (47.4%)	5 (25%)	0.098	6 (50%)	17 (37%)	0.41
Etiology (Alcohol)	20 (60.6%)	8 (32%)	0.031	22 (57.9%)	6 (30%)	0.043	10 (83.3%)	18 (39.1%)	0.006
On lactulose	33 (100%)	11 (44%)	<0.001	38 (100%)	6 (30%)	<0.001	12 (100%)	32 (69.6%)	<0.001
On PPI	16 (48.5%)	7 (28%)	0.114	17 (44.7%)	6 (30%)	0.275	7 (58.3%)	16 (34.8%)	0.137
Cytokine levels									
FABP2 peripheral (pg/ml)	486.1 ± 186.8	339.6 ± 169.1	0.003	480.5 ± 194	313.6 ± 135.7	0.001	518.1 ± 167.2	398.1 ± 192.2	0.039
LPS peripheral (pg/ml)	176 ± 84	167.4 ± 97.3	0.712	181.7 ± 87	154.4 ± 93	<0.001	224.2 ± 95.3	158.7 ± 83.5	0.03
IL-6 peripheral (pg/ml)	10.9 ± 15.6	6.7 ± 10.3	0.451	11 ± 15.9	5.5 ± 6.9	<0.001	28.8 ± 17.5	4 ± 5.5	<0.001
IL-8 peripheral (pg/ml)	38.6 ± 21.3	24.5 ± 14.1	0.003	35.7 ± 20.5	23.1 ± 14.2	<0.001	58.2 ± 21.1	24.4 ± 11.1	<0.001
FABP2 hepatic vein (pg/ml)	698.2 ± 470.2	391 ± 292.7	0.002	689.7 ± 475.7	330.4 ± 146.6	0.001	665.6 ± 424.6	539.8 ± 430.5	0.249
LPS hepatic vein (pg/ml)	104 ± 68.6	92.3 ± 55.5	0.525	101 ± 67.5	95.2 ± 54.9	0.793	115.7 ± 77.1	94.7 ± 59.1	0.291
IL-6 hepatic vein (pg/ml)	11.6 ± 22.6	7.8 ± 12.7	0.759	11.2 ± 21.2	7.5 ± 13.9	0.377	22.7 ± 33.3	6.6 ± 11.4	0.011
IL-8 hepatic vein (pg/ml)	16.1 ± 23.5	8.9 ± 11.6	0.517	15.3 ± 22.2	8.8 ± 12.2	0.454	23.3 ± 28.3	10.3 ± 15.8	0.053

SD – Standard deviation; MELD – Model of End-Stage Liver Disease; HVPG – Hepatic venous pressure gradient; PPI – Proton pump inhibitors; FABP2 – Fatty acid-binding protein 2; LPS – Lipopolysaccharide; IL – Interleukin.

Wilcoxon ranked-sum and Chi square tests were used to compare groups.

## Discussion

In this study, we aimed to evaluate circulating bacterial DNA signatures, inflammatory cytokine levels, and gut permeability markers in different blood compartments of patients with cirrhosis and PH. We identified significant clustering of circulating microbiome profiles between patients with cirrhosis and those of healthy controls. The differences could be explained by the differential abundance of several families and genera of bacteria, indicating a circulating microbiome shift in patients with cirrhosis. The relative abundance of *Escherichia*/*Shigella* and *Prevotella* was discriminant for liver cirrhosis versus that of the healthy controls, and together with *Bacteroides*, they were more abundant in patients with MELD scores >15 and in those with SPH. However, we did not find significant differences between the hepatic vein blood and peripheral vein blood microbiome profiles. *Escherichia/Shigella* and *Prevotella* showed significant correlations with clinical parameters and IL-8 concentrations in the hepatic vein. The circulating microbiome profile could not predict CSPH or SPH in our cohort of patients with cirrhosis.

Several previous studies have reported circulating microbiome profiles in patients with various liver diseases.^13–15,21,22^ Blood levels of *Enterobacteriaceae* are increased in the liver of patients with cirrhosis^15^ and are linked with mortality in hepatitis B-related liver failure.^13^ Consistent with these previous findings, we observed increased abundances of *Enterobacteriacea, Escherichia*/*Shigella*, and *Prevotella* in patients with cirrhosis. Schierwagen *et al*. recently reported discriminant microbial profiles in different human circulatory compartments of seven patients with liver cirrhosis, suggesting distinct genera in peripheral, hepatic, portal, and atrial blood.^21^ We detected no significant differences between hepatic vein blood and peripheral vein blood microbiome profiles, as measured by α-diversity or β-diversity; however, discrepancies between the study results need further evaluation.

We were unable to correlate intestinal and circulating taxa in the patients with cirrhosis, as fecal samples were not available for our cohort. Nevertheless, the relative similarity of previously reported stool microbial sequencing data in liver cirrhosis and the circulating microbial profiles in our current study suggest that the origin of the circulating microbiome may be linked to bacterial translocation from the gut.^23–26^ Previous studies that analyzed fecal samples showed that patients with cirrhosis display enrichment of gram-negative taxa, including members of the family *Enterobacteriaceae* and the genus *Bacteroides*,^26^ while *Prevotella* is associated with worse liver function.^23–26^ Furthermore, increased gut permeability and bacterial translocation in our cohort may have been indirectly supported by higher FABP2 levels, which have also been previously reported in liver cirrhosis.^27^ Changes in gut permeability may occur via different pathogenic pathways, including impaired mucous barrier, disruption of intestinal cells, damage of tight junctions, and altered innate pattern recognition receptors, among others.^28^ However, there is currently no effective biomarker for changes in gut permeability. Fatty acid-binding proteins (FABPs) are small cytosolic proteins found in mature enterocytes that are released after cell damage and serve as a biomarker of endothelial cell integrity.^29^ Unfortunately, FABPs are not ideal markers of intestinal permeability and may not reflect other permeability pathways, such as endothelial dysfunction or disruption of tight junctions between endothelial cells, which also occur in cirrhosis.^30^ This might explain why we failed to observe significant correlations between circulating microbiome signatures and FABP2.

Levels of proinflammatory cytokines IL-6 and IL-8, and LPS were significantly higher in our patients with cirrhosis than in the healthy controls, which is consistent with previous studies suggesting roles for endotoxemia and proinflammatory states in the development and progression of cirrhosis.^19^ Few previous studies have compared cytokine levels in different blood compartments and have reported somewhat contradicting results.^7,20,31^ For instance, the highest concentrations of IL-6 are found in the portal circulation, while hepatic vein levels increase in more decompensated patients, indicating diminished liver clearance of cytokines.^20^ The same study also reported that peripheral levels of IL-6 are higher than those found in the hepatic vein, possibly due to hemodynamic disturbances, such as portosystemic shunting.^20^ However, another study comparing the portal and hepatic blood compartments showed no differences in IL-6 or IL-8 levels.^31^ In our current study, we found significantly higher levels of LPS and IL-8 in the peripheral circulation when comparing compartments of patients with cirrhosis, but we observed no difference in IL-6 levels. The differences reported among the various studies need to be addressed in future large-scale studies that consider the entire spectrum of clinical variables.

We detected several bacteria in the hepatic vein blood that correlated with the inflammatory cytokine levels. *Escherichia/Shigella* and *Prevotella* exhibited the strongest correlations with IL-8. This may be explained by the ability of these genera of bacteria to produce LPS and promote inflammation via toll-like receptor (TLR) or inflammasome cascades.^32–34^ We also found a significant correlation between hepatic venous levels of IL-8 and *Ruminococcus*. In earlier studies, IL-8 receptor CXCR1 was found to be associated with hepatic inflammation,^35^ while *Ruminococcus* has been linked to liver fibrosis.^36^ Previously reported deregulated autochthonous taxa *Faecalibacterium* and *Roseburia*^37^ were also associated with increased levels of IL-8 in the hepatic blood of patients in our cohort. As *Escherichia/Shigella* and *Prevotella* were enriched in patients with SPH and those with MELD scores > 15 and correlated with IL-8 levels, we hypothesize that specific bacterial strains contribute to the development of cirrhosis by acting as proinflammatory triggers. Inflammasomes have been extensively studied in the pathogenesis of various diseases, including cirrhosis, but the microbiome, *per se*, cannot explain all inflammatory cascades associated with liver disease.^38,39^ Due to increased intestinal permeability and bacterial translocation in liver cirrhosis, an increased exposure to microbe-derived pathogen-associated molecular patterns may also play an important role in disease pathogenesis via the inflammasome activation cascade.^34,38–40^At the same time, the levels of circulating microbes appear to be very low in different blood compartments and the extent of this phenomenon might be very limited. Since gut microbes are detected at several-fold higher concentrations than those of the circulating microbiome, we can speculate that the gut microbiome may still be the leading source of microbiome-mediated inflammatory triggers in cirrhosis.^41^

The concept and diagnosis of CSPH is HVPG-driven, which cannot be completely replaced with noninvasive tools;^1^ however, different imaging and blood-based approaches have been evaluated as potential noninvasive biomarkers for the evaluation of PH in various clinical settings.^42^ While we were unable to find studies utilizing microbiome-based diagnostic tools as predictors of PH severity, recent studies have proposed the use of gut microbiome profiles as novel noninvasive biomarkers for liver diseases, such as NAFLD-cirrhosis, early stage hepatocellular carcinoma, and primary sclerosing cholangitis.^43–45^ A recent study published in *Nature* reported a circulating microbiome-based diagnostic tool that is able to discriminate cancer cases based on plasma-derived microbial profiles.^18^ In the current study, we could not identify a direct link between circulating microbiome profiles and PH as none of the bacterial genera in the hepatic or peripheral veins of patients with cirrhosis correlated with HVPG. This observation is indirectly in line with an important previous study that demonstrated that the treatment of patients with cirrhosis with rifaximin does not reduce the HVPG.^46^ Although *Bacteroides, Escherichia/Shigella*, and *Prevotella* were more abundant in patients with SPH in our current study, the absence of correlation between any genera and HVPG and poor AUC values challenge the value of the circulating microbiome to be used as a potential biomarker for PH.

Our data show that bacterial DNA can be detected in human plasma samples from healthy individuals. This suggests that certain gut bacteria may translocate via blood at any given time. We speculate that the immune system of healthy individuals is able to contain the translocating bacteria without causing any systemic effect. It is well known that symbiosis with intestinal microorganisms is important for tissue and immunity development^47^ and metabolic functions,^48^ and it provides protection against various pathogens.^49^ While several studies have linked circulating microbiome with diseases, such as alcoholic hepatitis,^14^ rheumatoid arthritis,^17^ and cardiovascular disorders,^50^ it remains difficult to define potentially risky strains in the circulating microbiome as only a limited number of studies have explored microbiome compositional changes in the blood.^12^ Based on the results of our current study and those of previously reported pathophysiological mechanisms,^51^ genera such as *Escherichia/Shigella* or *Prevotella* may act as TLR4 agonists or ligands for inflammasome activation and activate pathways leading to a pro-inflammatory state of cirrhosis.^34,40,52^

Our study had several limitations. First, we did not have paired fecal samples for our cohort and were unable to correlate the gut and circulating blood microbiome data. We were only able to compare our data with 16S sequencing data of fecal samples from previous liver cirrhosis studies. Having paired fecal samples would have been extremely valuable, and this important aspect of the puzzle needs to be addressed in future studies. Second, some of the patients in our cohort were on proton pump inhibitor (PPI) treatment at the time of inclusion in our study, which may have created a certain bias. It is not known whether PPIs can affect the circulating microbiome, but it has been shown that they have an important effect on the gut microbiome.^53^ Third, half of the patients in our cohort had alcohol-induced liver cirrhosis, but none had been active drinkers for at least 1 month prior to inclusion in the study. It is important to note that alcohol consumption may also have a modifying effect on microbial signatures.^54^

We also want to note that our study was a cross-sectional study with samples collected at a single time point. To the best of our knowledge, no data on circulating microbiome stability has currently been provided; however, it has been shown that the gut microbiome composition is prone to perturbations, especially during disturbing events such as infections.^55^ Nevertheless, longitudinal studies have also shown the compositional and functional stability of the gut microbiome over months and years; thus, evaluation of the microbiome at a single time point can also provide valuable information.^55,56^ A recent study demonstrated a relatively stable fecal microbiome in patients with stable cirrhosis, but the microbiome can be disturbed in cases of deteriorating disease.^57^ Although it would be extremely difficult to collect samples from different blood compartments at several time points, additional sample collection, especially during episodes of decompensation, would provide additional important information about the potential role of the circulating microbiome.

A major challenge in studies analyzing circulating microbiome DNA profiles is related to the potential contamination of low biomass samples at various stages of sample processing.^58,59^ However, accumulating data suggest that specific microbiome profiles are present in different human microenvironments.^59^ While approaches have been suggested to address contamination, currently available methods are unable to identify all contaminating taxa.^60^ To minimize the pitfalls related to the issue noted above, we determined any taxa sequences generated in sterile water control samples that underwent DNA extraction and PCR amplification prior to sequencing and eliminated those sequences from our analysis. The circulating microbiome signatures identified in our study appear to reflect prior results from stool microbiome studies regarding cirrhosis and indirectly indicate the biological relevance and potential intestinal origin of the circulating microbiome.^23–26^ However, methods for future analyses of circulating blood microbiome should be improved and better standardized.

In conclusion, we demonstrated a distinct circulating blood microbiome profile in patients with cirrhosis. We also showed that specific bacterial genera in the blood were marginally associated with SPH, MELD score, and inflammatory biomarkers. However, circulating microbial composition did not predict the severity of PH and probably will not be an efficient noninvasive marker for detecting CSPH or SPH.

## Materials and methods

**Figure 7.**
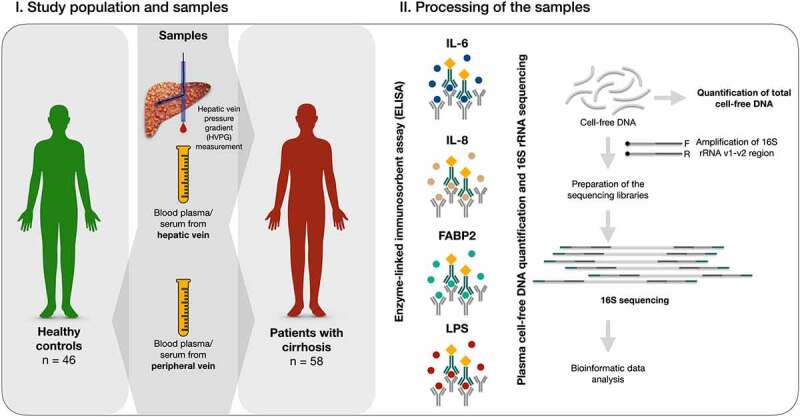
Workflow of the study.

The study included 58 consecutive outpatients with stable hepatitis C or alcohol-induced cirrhosis who were scheduled for a HVPG measurement. In addition, 46 healthy individuals were enrolled as controls. Demographic and clinical characteristics of the patients with cirrhosis, including CTP scores, MELD scores, PPIs, and lactulose use are shown in Table 1 and Table 2. All patients with cirrhosis underwent a scheduled HVPG measurement at the Department of Gastroenterology at the Lithuanian University of Health Sciences from September 2014 to December 2019. None of the patients with alcohol-induced cirrhosis had been active drinkers for at least 1 month prior to inclusion in the study. Cirrhosis was diagnosed according to standard clinical, laboratory, and radiologic criteria. Exclusion criteria for patients with liver cirrhosis were as follows: the presence of any other medical condition, including diabetes mellitus, cardiovascular disease, neurodegenerative conditions, acute kidney injury, hepatorenal syndrome, or cancer; active infection, including spontaneous bacterial peritonitis (SBP); portal vein or hepatic vein thrombosis; current use of beta-blockers or other vasoactive drugs; and use of antibiotics within 1 month prior to inclusion. Healthy control individuals were free of any chronic medical condition and had not received any medications during the previous 3 months prior to inclusion in the study. Demographic data, clinical parameters, liver function tests, and clinical chemistry data were collected from all participants at the time of inclusion in the study. The study protocol was approved by the Kaunas Region Biomedical Research Ethics Committee (2015–08-24, No. BE-2-28, Kaunas, Lithuania). All participants provided written informed consent prior to participating in the study. The study was conducted in accordance with the Declaration of Helsinki. A schematic of the workflow of the study is shown in Figure 7.

### HVPG measurement

All HVPG measurements were performed by the same experienced interventional radiologist according to the protocol described by Groszmann and Wongcharatrawee.^61^ At least three repeated measurements were performed for each patient to determine free and wedged hepatic vein pressures. HVPG values of 1–5 mmHg were considered to represent normal portal pressures, whereas PH was diagnosed at an HVPG ≥ 6 mmHg. An HVPG ≥ 10 mmHg was considered to be CSPH and an HVPG ≥ 12 mmHg was considered to be SPH.

### Blood sample collection, immunohistochemistry, isolation of nucleic acids, and sequencing

Peripheral blood samples were drawn from all subjects at the time of enrollment in the study. Blood samples were collected from the hepatic vein during the HVPG measurement procedure. Within 1 hour after drawing, the blood samples were placed at −80°C and stored until further processing.

A Human FABP2/I-FABP Quantikine ELISA Kit (DFBP20; R&D Systems), Human IL-6 Quantikine ELISA Kit (D6050; R&D Systems), Human IL-8/CXCL8 Quantikine ELISA Kit (D8000C, R&D Systems), and Human Lipopolysaccharides (LPS) ELISA Kit (CSB-E09945h; Cusabio) were used to quantify serum levels of FABP2, IL-6, IL-8, and LPS in cirrhosis patients and the healthy control subjects.

Circulating nucleic acids were extracted from blood plasma using a column-based QIAamp Circulating Nucleic Acid Isolation Kit (55114; Qiagen) according to the manufacturer’s protocol. Specific primer pair sets 27 F and 338 R targeting the hypervariable region V1-V2 were used in the polymerase chain reaction (PCR) process. Sequencing of the 16S ribosomal RNA gene was performed using the Illumina MiSeq platform (Illumina Inc.) and a dual-indexing approach. The acquired sequencing data were assigned into amplicon sequencing variants and taxonomically annotated against the RDP v16 database using the ‘dada2’ software package in R. A detailed description of the methods used for quantification of the FABP2, IL-6, IL-8, LPS, and 16S rRNA gene sequencing is provided in the Supplementary Materials.

### Statistical analysis

All statistical analyses were performed using the R programming environment. Nonparametric tests, including the Wilcoxon rank-sum test and the Kruskal-Wallis test, were used for routine statistical analysis where appropriate. Specific statistical analysis of the microbiome data was performed using freely accessible R packages, including phyloseq, vegan, DESeq2, and zinbwave.^62–65^ Only taxa meeting the criteria of a minimum abundance of two in one sample and present in at least 10% of the samples were included in the downstream analysis. The taxa present at >20% were included in differential abundance analysis. The Shannon index was used to measure α-diversity. Permutational analysis of variance (PERMANOVA) using the vegan package was used to detect significant changes in Bray-Curtis dissimilarity, as a measure of β-diversity. Differential abundance analysis was performed using the DESeq2 package, incorporating zero-inflation weights assessed by the zinbwave package. The results were controlled for age and gender as covariates. The discriminative power of the circulating microbiome profile was assessed using ROC curve analysis. Correlations between taxa abundances, clinical parameters of cirrhosis, inflammatory cytokines, and intestinal permeability markers were evaluated using Spearman’s correlation test. P-values were adjusted for multiple testing following the Benjamini–Hochberg procedure and p-values <0.05 were considered statistically significant.

## Supplementary Material

Supplemental Material

## Data Availability

Illumina reads of the 16S rRNA gene amplicon that support the findings of this study are available from the Open Science Framework repository at https://doi.org/10.17605/OSF.IO/T2ED7.

## List of abbreviations

PH – portal hypertension

MELD – Model of End-Stage Liver Disease

CTP – Child-Pugh-Turcotte

CSPH – Clinically significant portal hypertension

SPH - Severe portal hypertension

cfDNA - Cell-free DNA

LPS – Lipopolysaccharides

FABP2 – Fatty acid-binding protein 2
