# Supplementary material for: Circulating microbiome in patients with portal hypertension
Source: Gut Microbes. 2022 Feb 7;14(1):2029674. doi: 10.1080/19490976.2022.2029674 (PMC8824227; doi:10.1080/19490976.2022.2029674)
Supplement: Supplemental Material [file KGMI_A_2029674_SM1876.zip › supplementary/Supplementary Materials.docx]

**Supplementary Methods**

**Assessment of serum FABP2, IL-6, IL-8, LPS levels**

Serum levels of FABP2, IL-6, IL-8 and LPS were measured with enzyme-linked immunosorbent assay (ELISA) from patients’ serum. The ELISA kits used were: Human FABP2/I-FABP Quantikine ELISA Kit (DFBP20; R&D systems, MN, USA), Human IL-6 Quantikine ELISA KIT (D6050; R&D systems, MN, USA), Human IL-8/CXCL8 Quantikine ELISA Kit (D8000C, R&D systems, MN, USA), Human Lipopolysaccharides (LPS) ELISA Kit (CSB-E09945h; Cusabio, China). After optical density measurements at 450 nm and 570 nm (as the reference) wavelengths using Tecan Sunrise absorbance microplate reader, concentrations were calculated using four parameter logistic regression (4-PL) curve fitting model. The results were considered positive if they were higher than the cut-off levels recommended by the manufacturer.

**DNA extraction, 16s rRNA gene amplification and sequencing**

Peripheral blood was drawn prior to HVPG measurement, whereas hepatic vein blood samples were obtained during HVPG measurement procedure. Blood samples were collected in a sterile BD Vacutainer K_2_EDTA tubes (10 mL, 366643; Becton, Dickinson and Company, NJ, USA). Collected blood samples were centrifuged twice at room temperature by an optimized protocol: i) at 2000 x g for 10 minutes with a smooth brake profile to prevent disruption of the buffy coat; and ii) at 3000 x g for 10 minutes with a smooth brake profile to separate plasma. Plasma samples were aliquoted in Eppendorf Biopur® (Germany) single-packed 1.5ml tubes (certified for sterility, free of pyrogens, RNases, DNases, PCR inhibitors, free of human, bacterial DNA and ATP free) and then stored at -80 °C until further processing.

Total circulating nucleic acids from plasma were extracted using column-based QIAamp Circulating Nucleic Acid isolation kit (55114; Qiagen, Hilden, Germany) according to the manufacturers’ protocol. The isolated samples were stored at -20 °C until further analysis. Cell-free DNA (cfDNA) was quantified by laser-induced fluorescence-based microcapillary electrophoresis using TapeStation 2200 system (Agilent Technologies, CA, USA). High-Sensitivity D5000 ScreenTape and reagents (5067- 5592 and 5067- 5593; Agilent Technologies, CA, USA) were used for length distribution and concentration evaluation of isolated total cfDNA.

Isolated DNA from blood plasma samples was amplified targeting V1-V2 hypervariable region of bacterial 16S rRNA gene. Specific primer pair set 27F (AGAGTTTGATCCTGGCTCAG) and 338R (TGCTGCCTCCCGTAGGAGT) and dual-indexing was used in the process of PCR (cycling conditions: (98 °C, 30 s; 30 × [98 °C, 9 s; 55 °C, 60 s; 72 °C, 90 s]; 72 °C, 10 min; 10 °C, infinity). The resulting PCR products were purified and normalized utilizing the Invitrogen SequalPrep Normalization Plate Kit (Thermo Fisher Scientific, Waltham, USA). 16S rRNA gene sequencing was performed on Illumina MiSeq platform according to manufacturer’s instructions with MiSeq Reagent Kit v3.

DNA extraction blank controls were used to address for potential contamination in isolation reagents. A no template control was used as a PCR control that was set up in the same way as all other sample wells but did not contain any template DNA. It served as a general control for extraneous nucleic acid contamination. DNA extraction blank control samples underwent the same nucleic acid isolation procedure as all of the other sample wells, but instead of biologically meaningful sample, molecular grade water (Distilled, deionized, sterile-filtered water, DNase, RNase, and protease-free) was used in the workflow. DNA extraction blank controls and no template controls were sequenced as regular samples. Sequences obtained in these control samples were then removed from the downstream analysis as potential contaminants.

Acquired sequencing data was assigned into amplicon sequencing variants (ASV) using ‘dada2’ R package (V.1.10), following the DADA2 workflow (<http://benjjneb.github.io/dada2/tutorial.html>). Forward and reverse reads were trimmed, filtered and truncated to 200 and 150 nucleotides respectively. Reads with ambiguous bases and more than two expected errors were excluded from the workflow. Chimeric ASVs, singletons were removed. The remaining ASVs were taxonomically annotated according to the RDP database V16 using the naïve Bayesian classifier implemented in DADA2.

**Supplementary Figure S1. Circulating microbiome composition of the study cohort**


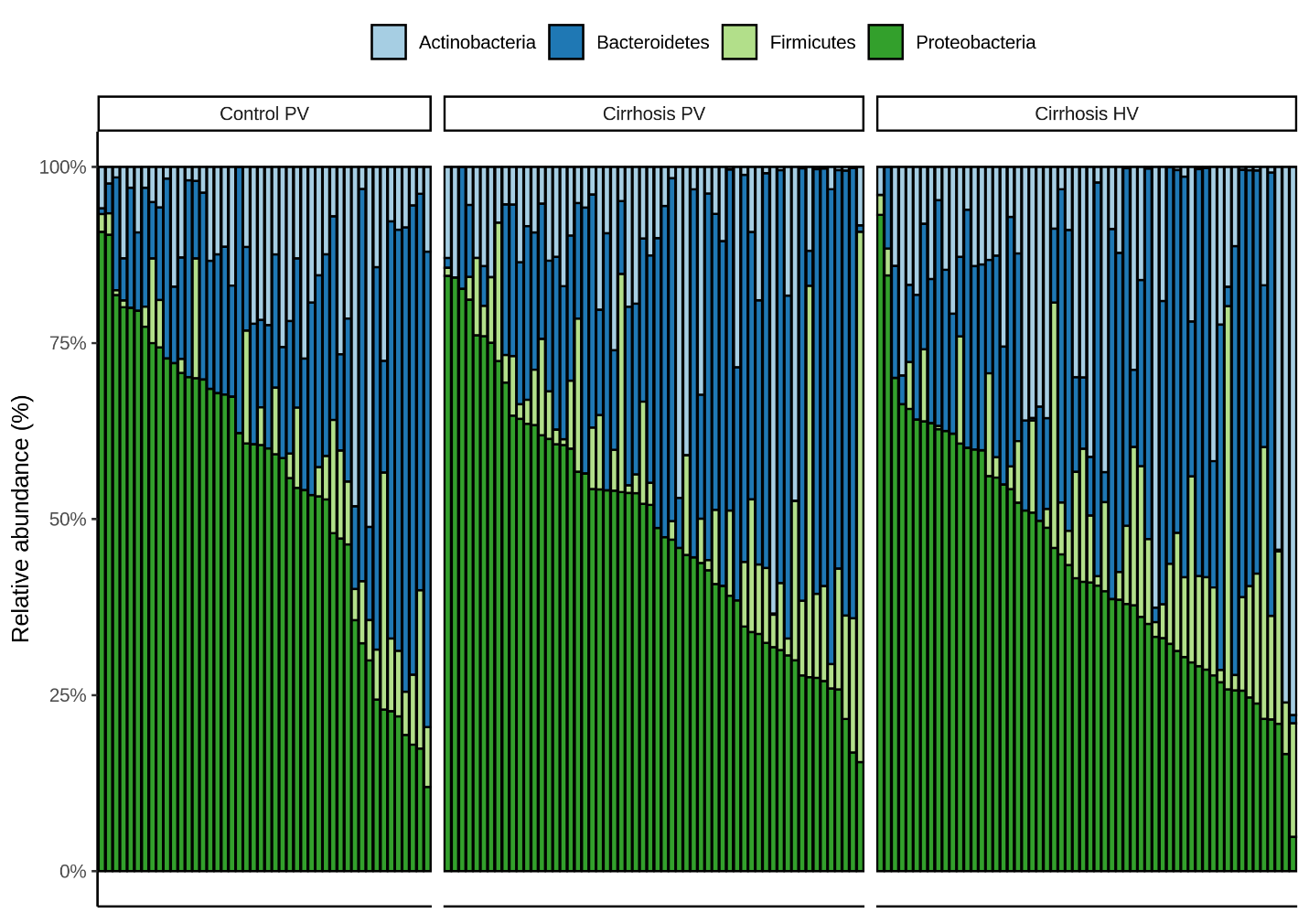


Bar plots representing phylum relative abundances in peripheral blood of individual liver cirrhosis patients and controls and hepatic vein blood of cirrhosis.

PV – Peripheral vein; HV – Hepatic vein

**Supplementary Figure S2. Circulating microbiome composition in the peripheral vein of patients with cirrhosis and controls**


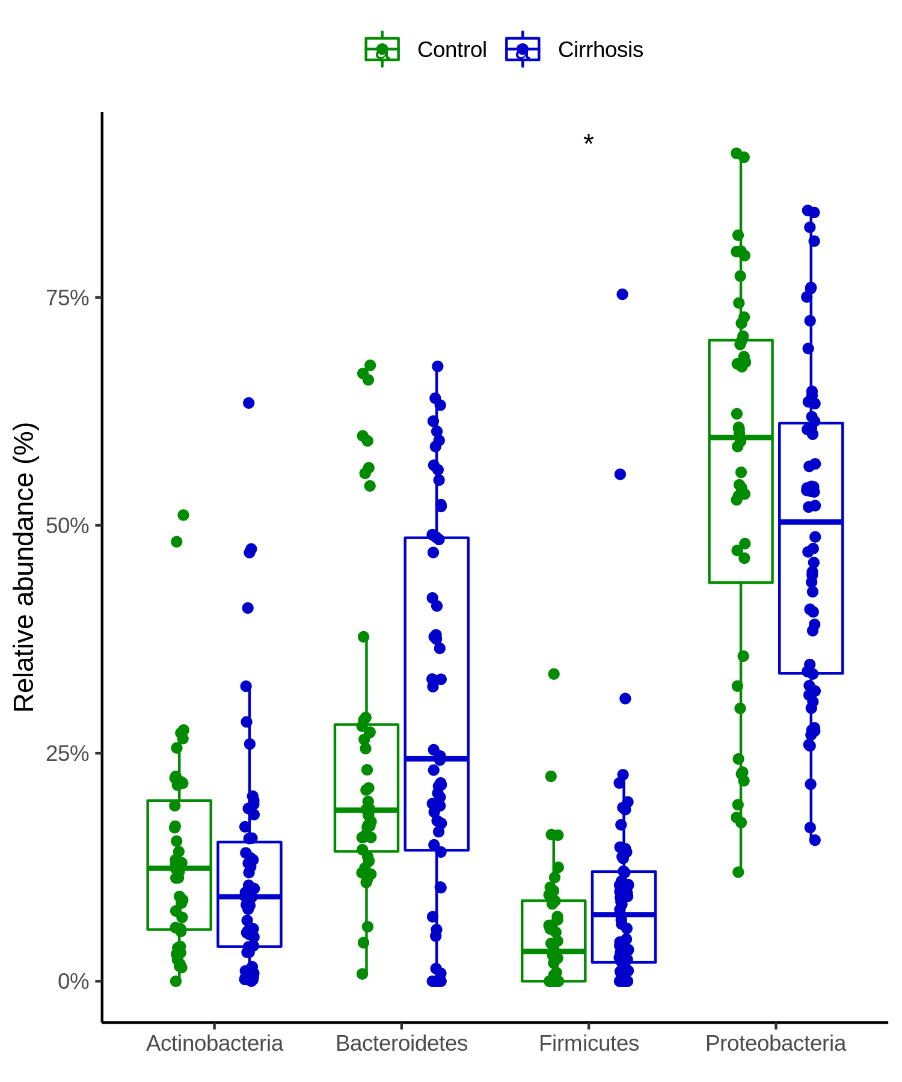


Boxplots represent minimum, first quartile, median, third quartile, and maximum values of the relative abundances. Kruskal Wallis test was used to compare groups. * indicates significant differences between groups. * - *P < 0.05*

**Supplementary Figure S3.** Differentialy abundant genera between controls and patients with cirrhosis


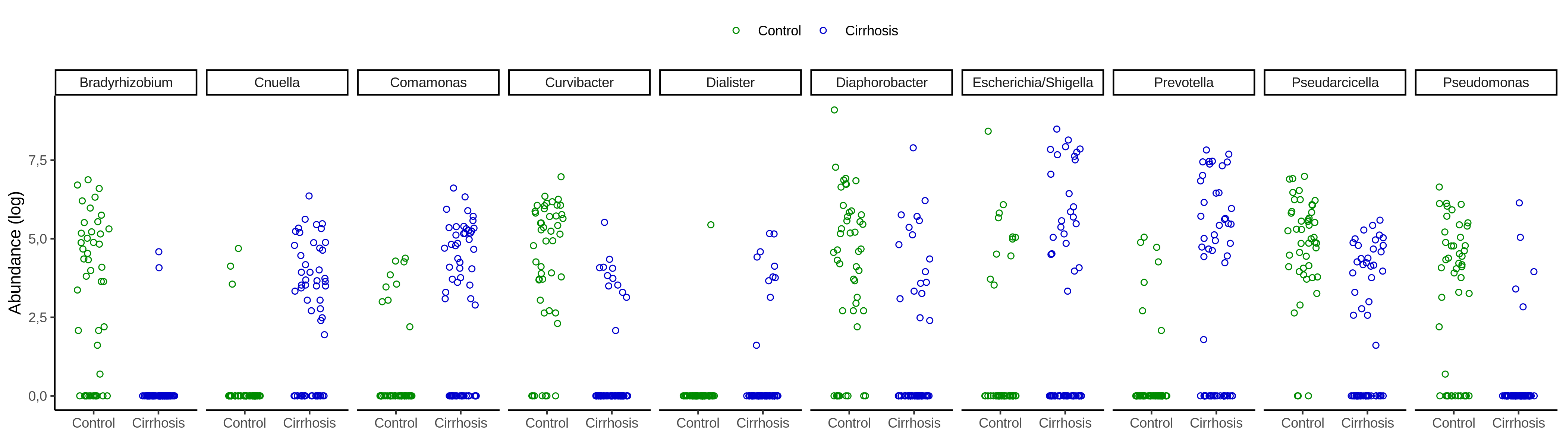


Strip chart showing peripheral vein log-transformed count abundance of differentialy abundant genera between controls and patients with cirrhosis.

**Supplementary Figure S4. Peripheral levels of FABP2, LPS, IL-6 and IL-8 in patients with cirrhosis and controls**


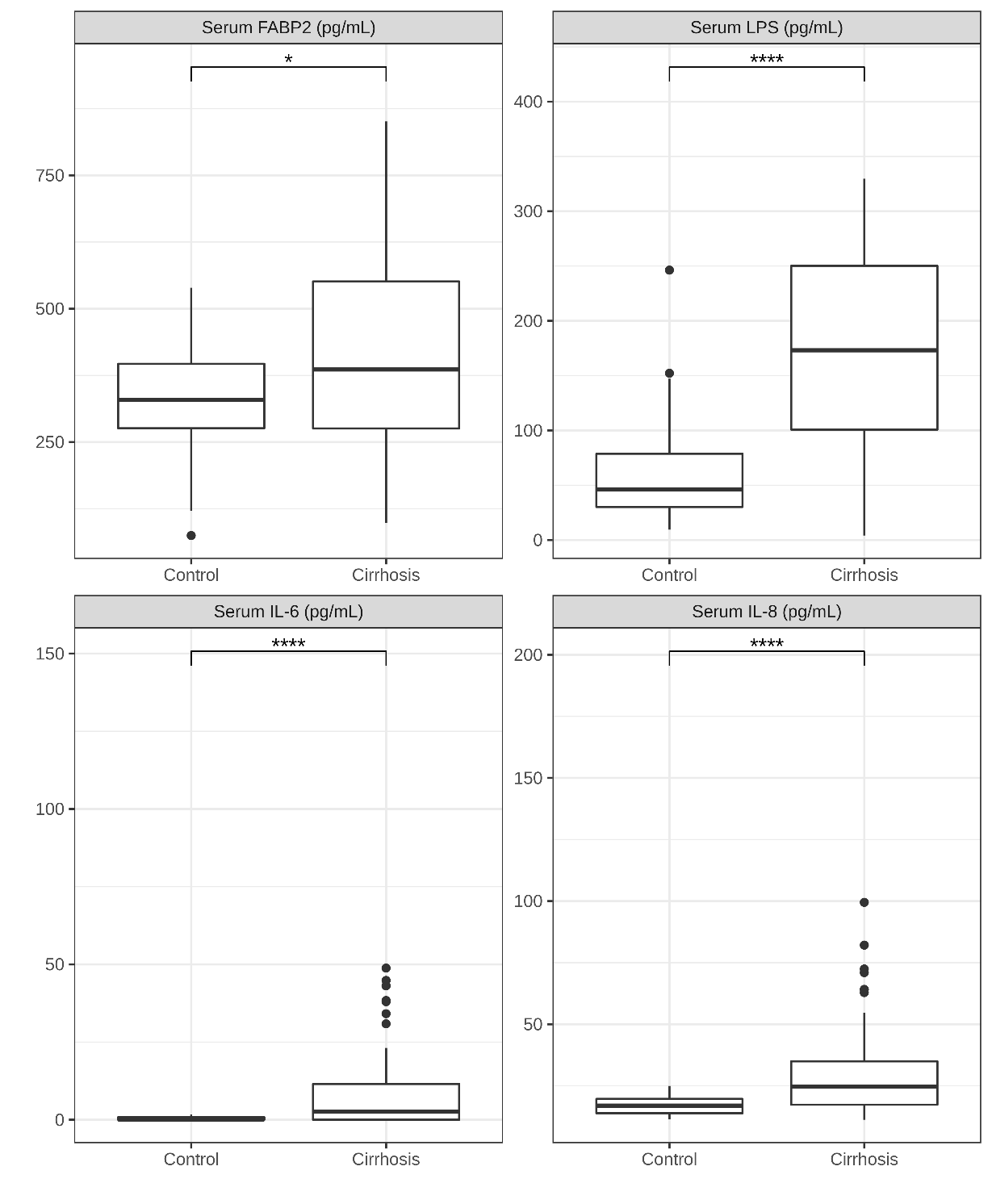

Boxplots represent minimum, first quartile, median, third quartile, and maximum values. Wilcoxon ranked-sum test was used to compare groups. * indicates significant differences between groups. * - *P < 0.05,* ** - *P < 0.01,* *** - *P* < 0.001; **** - *P* < 0.0001

**Supplementary Figure S5.** Abundances of genera *Bacteroides, Escherichia/Shigella* and *Prevotella* in sub groups of patients with liver cirrhosis in different blood compartments


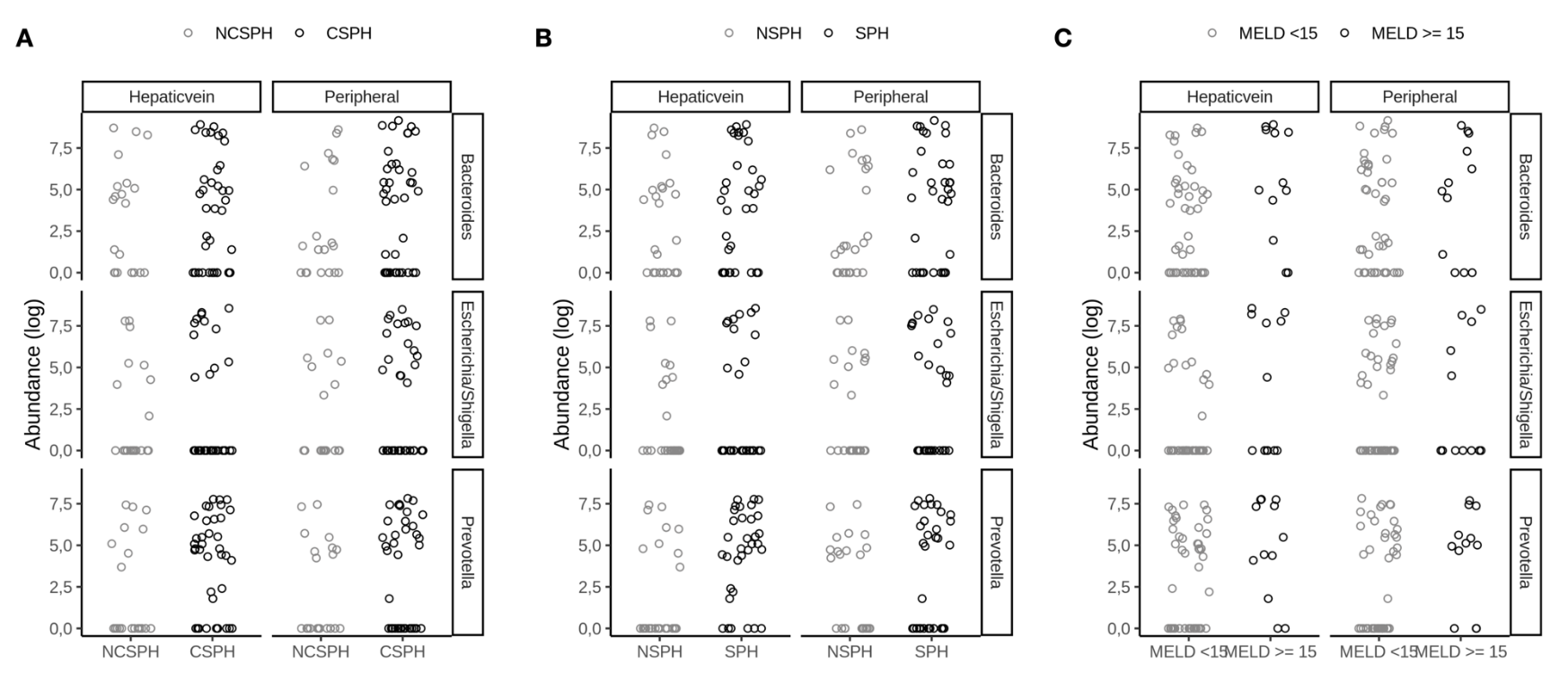


Strip chart showing log-transformed count abundance of genera *Bacteroides, Escherichia/Shigella* and *Prevotella* in different compartments of A) patients with NCSPH and CSPH; B) patients with NSPH and SPH; C) patients with MELD score < 15 and ≥ 15.

NCSPH - Not clinically significant portal hypertension; CSPH - Clinically significant portal hypertension; NSPH – Not severe portal hypertension; SPH - Severe portal hypertension; MELD - Model of End-Stage Liver Disease

**Supplementary Figure S6. Levels of FABP2, LPS, IL-6 and IL-8 in different blood compartments of patients with cirrhosis**


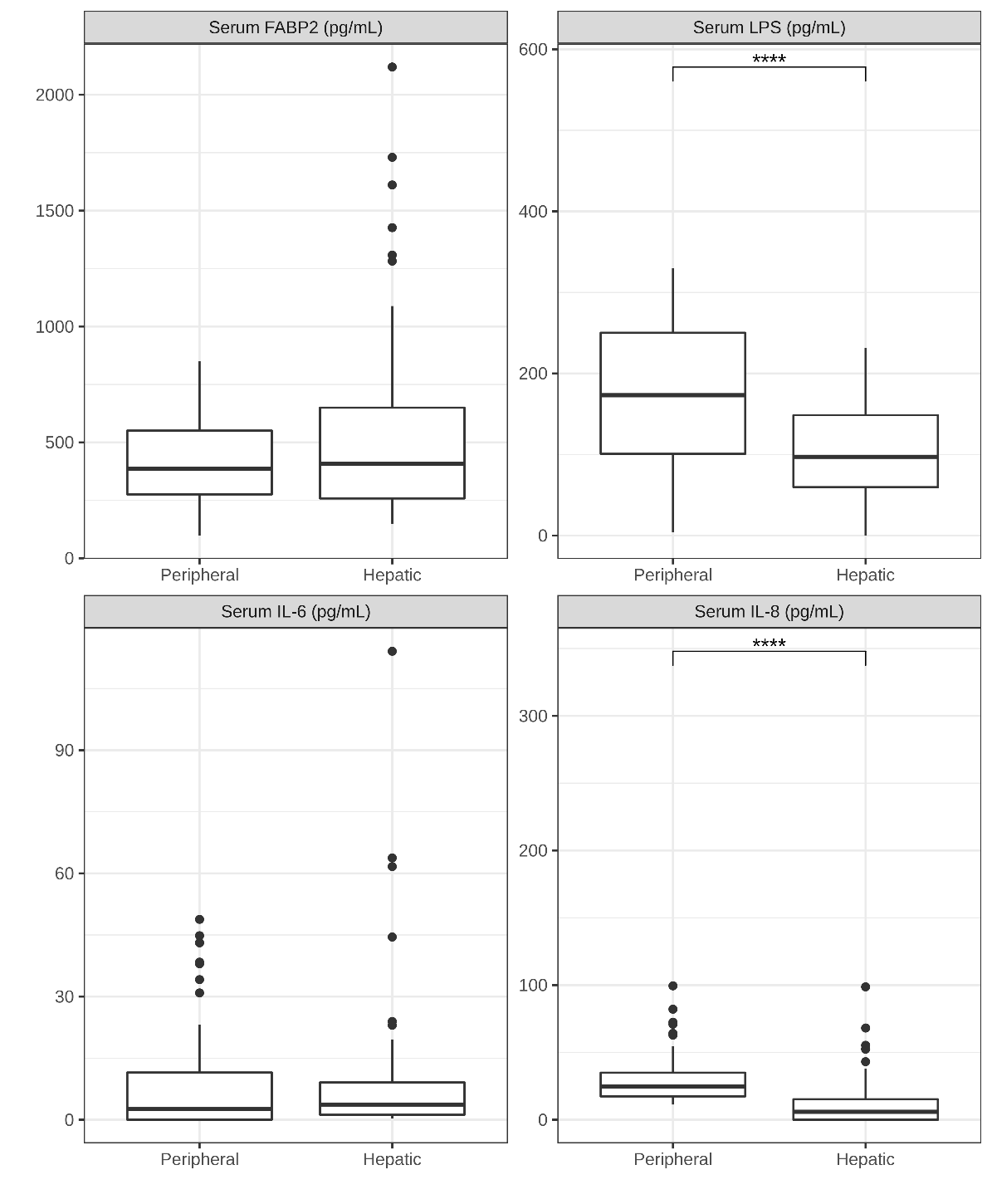

Boxplots represent minimum, first quartile, median, third quartile, and maximum values. Wilcoxon ranked-sum test was used to compare groups. * indicates significant differences between groups. * - *P < 0.05,* ** - *P < 0.01,* *** - *P* < 0.001; **** - *P* < 0.0001

**Supplementary Table S1.** Correlation between levels of LPS, IL-6, IL-8 and FABP2 in different blood compartments and clinical parameters of patients with cirrhosis.

|  | **Peripheral / Hepatic vein blood** | | |
| --- | --- | --- | --- |
|  | MELD | CTP | HVPG |
| LPS | ns / ns | ns / ns | ns / ns |
| IL-6 | 0.56 / ns | 0.613 / 0.351 | ns / ns |
| IL-8 | 0.698 / ns | 0.737 / ns | 0.378 / ns |
| FABP2 | 0.353 / ns | 0.302 / ns | 0.362 / 0.325 |

HVPG – hepatic venous pressure gradient; CTP – Child-Turcotte-Pugh; MELD – Model for End-Stage Liver Disease; ns – non-significant correlations. Numbers represent Spearman rank correlation coefficient at *P* value <0.05. Correlations between cytokine levels and clinical parameters in different compartments are separated by */* symbol.
